# British Journal of Dental Science

**Published:** 1874-04

**Authors:** 


THE BRITISH JOURNAL OF DENTAL SCIENCE. *Established sixteen years.* A monthly chronicle of current intelligence, relating to every Department of Dentistry and the collateral sciences, published on the first of each month.

It may be had of the American Agents, Lindsay & Blakiston, Philadelphia, at $4.00 per year, or 50 cents a copy.

